# (μ-Di-*tert*-butyl­silanediolato)bis­[bis­(η^5^-cyclo­penta­dien­yl)methyl­zirconium]

**DOI:** 10.1107/S2056989019014762

**Published:** 2019-11-08

**Authors:** David J. Berg, Leah Gajecki, Hunter Hill, Brendan Twamley

**Affiliations:** a Department of Chemistry, University of Victoria, PO Box 1700 Stn CSC, Victoria, BC V8W 2Y2, Canada; b School of Chemistry, Trinity College Dublin, University of Dublin, Dublin 2, Ireland

**Keywords:** crystal structure, siloxide, zirconium, metallocene, organometallic

## Abstract

The reaction of *t*-Bu_2_Si(OH)_2_ with two equivalents of Cp_2_Zr(CH_3_)_2_ produces the title *t*-Bu_2_SiO_2_-siloxide bridged dimer, [Zr_2_(CH_3_)_2_(C_5_H_5_)_4_(C_8_H_18_O_2_Si)] or [Cp_2_Zr(CH_3_)]2[μ-*t*-Bu_2_SiO_2_] (**1**), where one methyl group is retained per zirconium atom. The same product is obtained at room temperature even when equimolar ratios of the silanediol and Cp_2_Zr(CH_3_)_2_ are used. Attempts to thermally eliminate methane and produce a bridging methyl­ene complex resulted in decomposition. The crystal structure of **1** displays typical Zr—CH_3_ and Zr—O distances but the Si—O distance [1.628 (2) Å] and O—Si—O angle [110.86 (15)°] are among the largest observed in this family of compounds suggesting steric crowding between the *t*-Bu substituents of the silicon atom and the cyclo­penta­dienyl groups. The silicon atom lies on a crystallographic twofold axis and both Cp rings are disordered over two orientations of equal occupancy.

## Abstract 2

The reaction of *t*-Bu_2_Si(OH)_2_ with two equivalents of Cp_2_Zr(CH_3_)_2_ produces the *t*-Bu_2_SiO_2_-siloxide bridged dimer where one methyl group is retained per zirconium atom. The retention of one methyl group per metal center affords a site for further reactivity.

## Chemical context   

Zirconocene siloxides have been investigated for their ability to bond reactive metal centers to solid glass supports (Samuel *et al.*, 1994 ▸) and as potential precursors to novel inorganic polymers by cyclic siloxane ring-opening polymerization (Thieme *et al.*, 2002 ▸). In both of these examples, two diorganosilicon dioxide (μ-*R*_2_SiO_2_^2−^) ligands span two zirconocene units in a cyclic dimer. In contrast, the structure of the title compound **1**, shows only one bridging di-*tert*-butyl­silicon dioxide ligand and each zirconocene unit retains one reactive methyl group. The same product is obtained regardless of whether one or two equivalents of Cp_2_Zr(CH_3_)_2_ are used per equivalent of silanediol at room temperature. At higher temperatures, the NMR of the reaction mixture becomes more complicated but we were unable to cleanly obtain the cyclic equivalent of the compounds mentioned above, [Cp_2_Zr]_2_[μ-*t*-Bu_2_SiO_2_]_2_. This compound could potentially serve as an olefin polymerization pre-catalyst by methyl abstraction with [Ph_3_C]^+^[B(C_6_F_5_)_4_]^−^ or similar activators (see for *e.g.*, Babushkin *et al.*, 2014 ▸). Initial attempts to thermally eliminate methane and form a bridging methyl­ene complex, [Cp_2_Zr]_2_[μ-*t*-Bu_2_SiO_2_][μ-CH_2_], led to decomposition. 
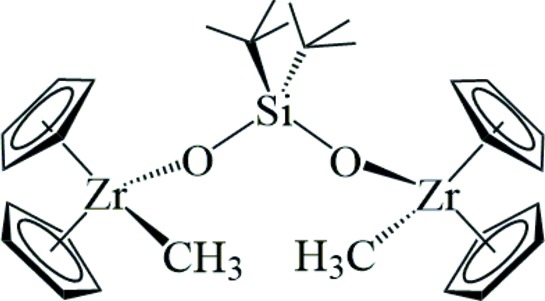


## Structural commentary   

**Figure 1:**
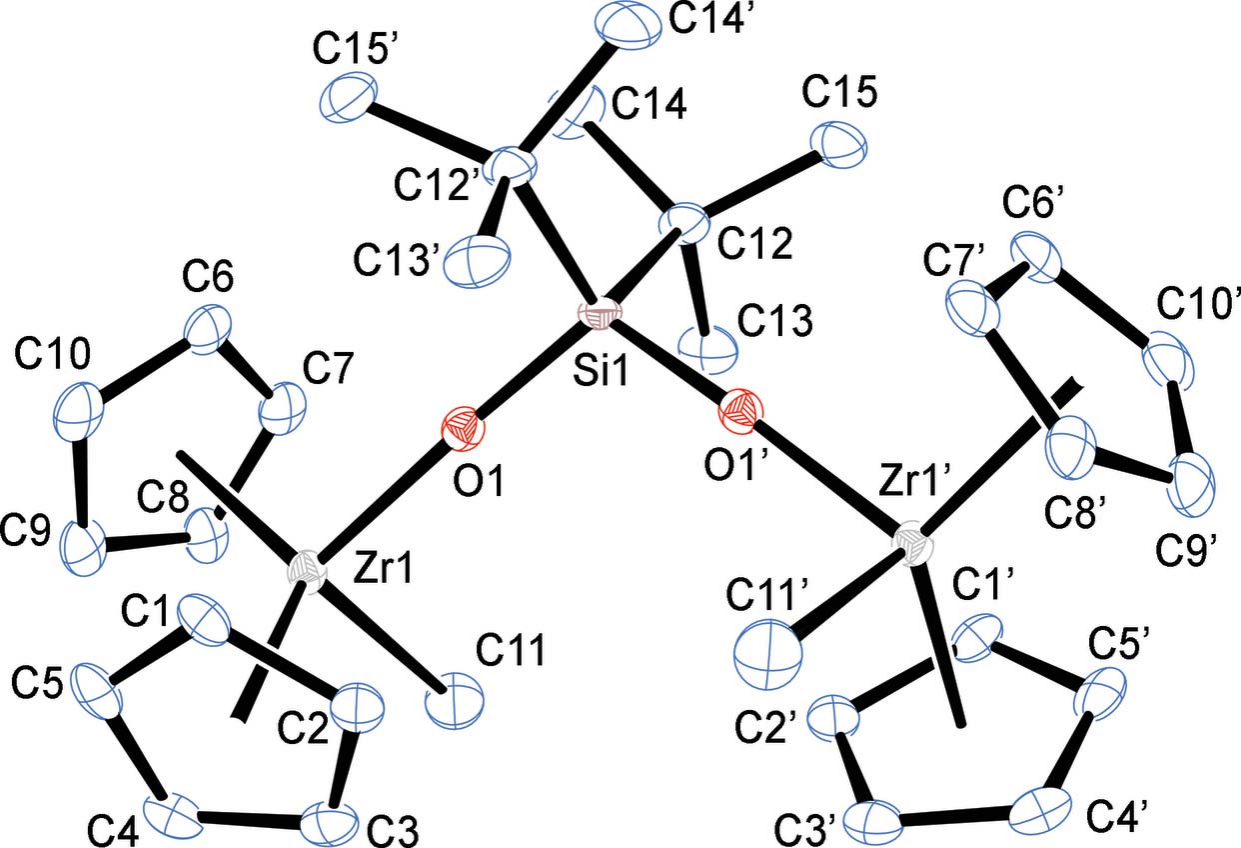
The mol­ecular structure of **1** with displacement ellipsoids drawn at the 50% probability level; hydrogen atoms omitted for clarity.

**Figure 2:**
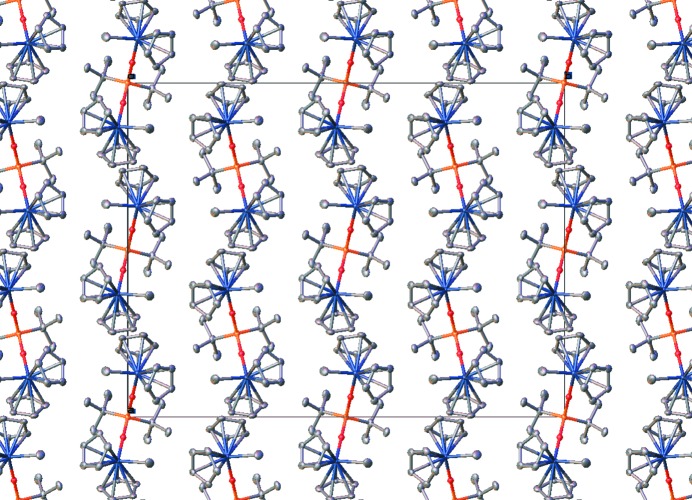
Packing diagram for one disorder partner of **1**, viewed down the *c* axis.

The mol­ecular structure of **1** is shown in Fig. 1 ▸ and a packing diagram is given in Fig. 2 ▸. The cyclo­penta­dienyl groups on Zr1 are both disordered and were modelled over two positions with 50% occupancy each. The diagrams in Figs. 1 ▸ and 2 ▸ show only one of the two disordered cyclo­penta­dienyl positions.

The Zr1—CH_3_ (C11) distance in **1** of 2.307 (3) Å is typical of other zirconocene methyl complexes (range: 2.24–2.39 Å, median: 2.29 Å). The Zr1—O1 and O1—Si1 distances of 1.960 (2) and 1.628 (2) Å, respectively, are typical of other zirconocene siloxides, although the latter distance is at the long end of the observed range (Zr—O range: 1.94–2.01 Å, median: 1.98 Å; Si—O range: 1.56–1.65 Å, median: 1.61 Å). The O1—Si1—O1(1 − *x*, −*y*, *z*) angle is 110.86 (15)°, which is the widest yet observed in an *R*_2_SiO_2_ bridged transition metal dimer (range: 103.7—110.2°). The wider O—Si—O angle and longer Si—O bond likely reflect increased steric crowding between the *t*-butyl substituents on Si and the Cp rings on Zr. Other key geometrical data are listed in Table 1 ▸.

## Supra­molecular features   

Assuming that they are not artifacts of disorder, there are some short inter­molecular π–π contacts between the Cp rings [shortest centroid–centroid separation = 3.862 (8) Å]. Otherwise, there are no exceptional features in the packing of **1**.

## Database survey   

There are 60 structures in the CSD (November 2018 version; Groom *et al.*, 2016 ▸) containing zirconocene units bonded to an anionic oxygen atom and a methyl group, Cp_2_Zr(CH_3_)(O*X*), that were used to compare the Zr—CH_3_ distance in **1**. Many of these structures contain a bridging oxo (O^2−^) group bridged to another metal, which is obviously quite different than the siloxide in **1**. A smaller subset of this group (19 structures) contain simple alkoxides as the anionic oxygen unit [*i.e.* Cp_2_Zr(CH_3_)(O*R*)]. If the comparison is restricted to just the latter structures, the Zr—C bond length range is somewhat narrower from 2.26–2.33 Å with a median of 2.29 Å (Bestgen *et al.*, 2016 ▸; Black *et al.*, 2008 ▸; Breen & Stephan, 1996 ▸; Chapman *et al.*, 2012 ▸; Frömel *et al.*, 2013 ▸; Gambarotta *et al.*, 1985 ▸; Jian *et al.*, 2018 ▸; Koch *et al.*, 2000 ▸; Mariott & Chen, 2005 ▸; Matchett *et al.*, 1988 ▸; Normand *et al.*, 2016 ▸; Stuhldreier *et al.*, 2000 ▸). There are 15 structures containing siloxide ligands bonded to a zirconocene unit in a pseudo-tetra­hedral environment used to compare Zr—O and O—Si distances in **1**. Of those structures, there are nine that contain simple siloxides that are not part of a polysiloxane cluster or a chelate ring system; using those structures for comparison results in no substantial change in the range or median Si—O or Zr—O bond lengths (Abrahams *et al.*, 1996 ▸; Burlakov *et al.*, 2006 ▸; Enders *et al.*, 2001 ▸; Hofmann *et al.*, 2002 ▸; Richers *et al.*, 2017 ▸; Samuel *et al.*, 1994 ▸; Thieme *et al.*, 2002 ▸; Zhang *et al.*, 2009 ▸). In addition, there are 14 structures containing the O_2_Si—*t*-Bu_2_ unit bridging two transition metals that were used for comparison to the O—Si—O angles in **1**. These structures include Ti (six structures: Haoudi-Mazzah *et al.*, 1991 ▸; Liu, Schmidt *et al.*, 1992 ▸; Liu, Roesky *et al.*, 1992 ▸; Liu *et al.*, 1995 ▸), Zr (Haoudi-Mazzah *et al.*, 1991 ▸), Hf (Liu *et al.*, 1996 ▸), V (Gosink *et al.*, 1993 ▸), Nb (Gosink *et al.*, 1994 ▸), Mo (Gosink *et al.*, 1993 ▸), W (Gosink *et al.*, 1994 ▸), Re (two structures: Roesky, Mazzah, *et al.*, 1991 ▸; Roesky, Hesse *et al.*, 1991 ▸).

**Crystal structures with Cp_2_Zr—CH_3_ units for Zr—C distance comparisons:**

AQESIZ (Mukherjee *et al.*, 2011 ▸); AXIBOA (Boulho *et al.*2016 ▸); BESGOW (Bolig & Chen, 2004 ▸); BODMIR (Helmstedt *et al.*, 2008 ▸); BUHVAD (Xu *et al.*, 2015 ▸); BUYSOD10 (Longato *et al.*, 1985 ▸); CADRUU (Hunter *et al.*, 1983 ▸); COHTEY (Waymouth *et al.*, 1984 ▸); COPRII (Ho *et al.*, 1984 ▸); DAGKAX and DAGKIF (Gambarotta *et al.*, 1985 ▸); DITHAP (Martin *et al.*, 1985 ▸); EHEFUT (Neu *et al.*, 2011 ▸); EKEVEX, EKEVIB, EKEVOH, EKEVUN, EKEWAU and EKEWEY (Normand *et al.*, 2016 ▸); ESISAA (Zuccaccia *et al.*, 2004 ▸); GIPYUZ (Matchett *et al.*, 1988 ▸); HEMCOR (Askham *et al.*, 1994 ▸); HIKHUF and HIKJAN (Gurubasavaraj *et al.*, 2007 ▸); HUVLAL (Fujdala *et al.*, 2003 ▸); IGUDOD (Hüerländer *et al.*, 2002 ▸); JITVAK and JITVEO (Mandal *et al.*, 2007 ▸); JUGCIZ (Boulho *et al.*, 2015 ▸); KEXYER (Erker *et al.*, 1990 ▸); KODQAV (Koch *et al.*, 2000 ▸); KUPQAP (Mariott & Chen, 2005 ▸); LEDBEB (Askham *et al.*, 1993 ▸); LEPXAH (Mukherjee *et al.*, 2013 ▸); MOJHEZ (Black *et al.*, 2008 ▸); NAHYOL (Bai *et al.*, 2005 ▸); NAPXUY (Pineda *et al.*, 2005 ▸); NIMNOM (Johnson *et al.*, 1997 ▸); ODOBIU, ODOBOA and ODOBUG (Frömel *et al.*, 2013 ▸); OKUFUX (Bestgen *et al.*, 2016 ▸); OZUCAO (Kelsen *et al.*, 2011 ▸); PEDFUA (Singh *et al.*, 2006 ▸); QIZCEI (Yang, Gurubasavaraj *et al.*, 2008 ▸); REDTUQ (Cummings *et al.*, 2006 ▸); TIWKUG (Yang, Schulz *et al.*, 2008 ▸); TOWMUN (Breen & Stephan, 1996 ▸); VIBSOO (Waymouth *et al.*, 1990 ▸); WAJLOJ (Ruck & Bergman, 2004 ▸); WATSOB, WATSUH and WATTAO (Chapman *et al.*, 2012 ▸); WAYMER (Liu *et al.*, (2017 ▸); WETJEL (Helmstedt *et al.*, 2006 ▸); WEWRUO (Jian *et al.*, 2018 ▸); WEXWED (Nekoueishahraki *et al.*, 2009 ▸); WUPVUA (Gurubasavaraj (2015 ▸); XESDEE Stuhldreier *et al.*, 2000 ▸); YIMKAG (Ciruelo *et al.*, 1995 ▸).

**Crystal structures with Cp_2_Zr–O–Si units for Zr—O and O—Si distance comparisons:**

EXUBII (Garrison *et al.*, 2004 ▸); HECZEU (Samuel *et al.*, 1994 ▸); JANYEF (Richers *et al.*, 2017 ▸); LEJSEZ (Burlakov *et al.*, 2006 ▸); QAMLEW (Wada *et al.*, 2004 ▸); REWKIN (Abrahams *et al.*, 1996 ▸); ROCWIP (Enders *et al.*, 2001 ▸); TUDQEP (Zhang *et al.*, 2009 ▸); UGINIH and UGINON; UMOWUO (Lacroix *et al.*, 2003 ▸); VAQMEH (Varga *et al.*, 2012 ▸); WUSWAI and WUSWEM (Thieme *et al.*, 2002 ▸); XIXDIR (Skowronska-Ptasinska *et al.*, 2001 ▸).

**Crystal structures with** ***M*** **–O–Si(** ***t*** **-Bu)_2_–O–** ***M*** **units for O—Si—O angle comparisons:**

HETRED and HETRON (Gosink *et al.*, 1994 ▸); JIYBEY (Roesky, Hesse *et al.*, 1991 ▸); KIPGUL (Roesky, Mazzah *et al.*, 1991 ▸); NADDAX (Liu *et al.*, 1996 ▸); PAHZED (Liu, Schmidt *et al.*, 1992 ▸); TAJYOS, TAJYUY and TAJZAF (Haoudi-Mazzah *et al.*, 1991 ▸); VUMNUM (Liu, Roesky *et al.*, 1992 ▸); WAGVIJ and WAGVOP (Gosink *et al.*, 1993 ▸); ZEKKAB and ZEKKEF (Liu *et al.*, 1995 ▸).

## Synthesis and crystallization   

**General.** All solvents were purchased from Sigma–Aldrich Chemicals and dried by distillation from sodium under nitro­gen. Cp_2_Zr(CH_3_)_2_ was purchased from Sigma–Aldrich Chemicals and used as received. Di-*t*-butyl­silanediol was prepared by the oxidation of *t*-Bu_2_Si(H)Cl (Sigma–Aldrich) with aqueous KMnO_4_ following the procedure of Lickiss & Lucas (1996 ▸). NMR spectra were recorded on a Bruker AVIII 300 MHz Spectrometer in sealable Teflon-valved tube and were referenced to residual solvent resonances. Elemental analyses were performed by Canadian Microanalytical Ltd.

**Figure 3:**
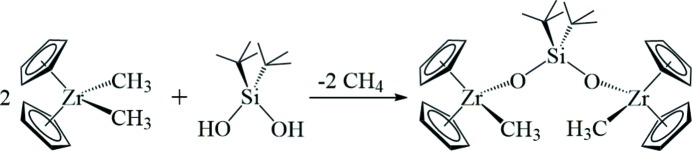
Reaction scheme.

**Synthesis.** The title compound was prepared (Fig. 3 ▸) by adding a toluene solution (5 ml) of di-*t*-butyl­silanediol (0.080 g, 0.45 mmol) to a stirred solution of di­methyl­zirconocene, Cp_2_Zr(CH_3_)_2_ (0.228 g, 0.907 mmol), in toluene (5 ml) in a 50 ml Erlenmyer flask in an inert atmosphere glovebox. After stirring overnight, the solution was concentrated under vacuum, layered with hexane and stored in a 243 K freezer. Large, colourless crystals of **1** deposited within a few days. Yield: 0.196 g (67%). ^1^H NMR (C_6_D_6_, 300 MHz): δ 5.905 (*s*, 20H, Cp*H*), 1.091 [*s*, 18H, C(C*H_3_*)_3_], 0.465 (*s*, 6H, C*H_3_*); ^13^C{^1^H} NMR (C_6_D_6_, 125 MHz): δ 111.32 (Cp*C*), 28.92 (C(*C*H_3_)_3_), 22.63 (*C*H_3_); *C*(CH_3_)_3_ not observed. Analysis calculated for C_30_H_44_O_2_SiZr_2_ (%): C, 55.68; H, 6.85. Found: C, 55.33; H, 6.71.

## Refinement   

Crystal data, data collection and structure refinement details are summarized in Table 2 ▸. Both Cp rings were found to be disordered and modelled over two sets of sites with 50% occupancy with restraints (SIMU cards). H atoms were positioned geometrically and refined as riding, with C—H = 0.95–0.98 Å and *U*_iso_(H = 1.2*U*_eq_(C) or 1.5*U*_eq_(C-meth­yl).

## Supplementary Material

Crystal structure: contains datablock(s) I. DOI: 10.1107/S2056989019014762/hb4319sup1.cif

Structure factors: contains datablock(s) I. DOI: 10.1107/S2056989019014762/hb4319Isup2.hkl

CSD refcodes and references for structures used for comparisons. DOI: 10.1107/S2056989019014762/hb4319sup3.docx

CCDC references: 1962802, 1962802

Additional supporting information:  crystallographic information; 3D view; checkCIF report

## Figures and Tables

**Table 1:** Selected geometric parameters (Å, °)

Zr1—Cp1	2.196	Zr1—Cp2	2.202
Zr1—Cp1′	2.258	Zr1—Cp2′	2.233
			
O1—Zr1—C11	98.83 (11)	C11—Zr1—Cp1′	96.81
O1—Zr1—Cp1	108.60	C11—Zr1—Cp2	98.16
O1—Zr1—Cp1′	109.30	C11—Zr1—Cp2′	106.27
O1—Zr1—Cp2	107.31	Cp1—Zr1—Cp2	130.26
O1—Zr1—Cp2′	110.27	Cp1′—Zr1—Cp2′	129.80
C11—Zr1—Cp1	109.36		

**Table 2:** Experimental details

Crystal data	
Chemical formula	[Zr_2_(CH_3_)_2_(C_5_H_5_)_4_(C_8_H_18_O_2_Si)]
*M*_r_	647.18
Crystal system, space group	Orthorhombic, *F* *d* *d*2
Temperature (K)	83
*a*, *b*, *c* (Å)	21.673 (4), 28.296 (6), 9.7466 (19)
*V* (Å^3^)	5977 (2)
*Z*	8
Radiation type	Mo *K*α
μ (mm^−1^)	0.76
Crystal size (mm)	0.35 × 0.27 × 0.17

Data collection	
Diffractometer	Bruker P4
Absorption correction	Multi-scan (*SADABS*; Bruker, 2002 ▸)
*T*_min_, *T*_max_	0.777, 0.882
No. of measured, independent and observed [*I* > 2σ(*I*)] reflections	18986, 4237, 4172
*R*_int_	0.025
(sin θ/λ)_max_ (Å^−1^)	0.705

Refinement	
*R*[*F* ^2^ > 2σ(*F* ^2^)], *wR*(*F* ^2^), *S*	0.024, 0.055, 1.12
No. of reflections	4237
No. of parameters	253
No. of restraints	319
H-atom treatment	H-atom parameters constrained
Δρ_max_, Δρ_min_ (e Å^−3^)	0.52, −0.53
Absolute structure	Flack *x* determined using 1892 quotients [(*I* ^+^)−(*I* ^−^)]/[(*I* ^+^)+(*I* ^−^)] (Parsons *et al.*, 2013 ▸)
Absolute structure parameter	−0.001 (16)

Computer programs: *SMART* and *SAINT* (Bruker, 2002 ▸), *SHELXS* (Sheldrick, 2008 ▸), *SHELXL* (Sheldrick, 2015 ▸) and *OLEX2* (Dolomanov *et al.*, 2009 ▸).

## supplementary crystallographic information

### Crystal data

**Table:**

[Zr_2_(CH_3_)_2_(C_5_H_5_)_4_(C_8_H_18_O_2_Si)]	*D*_x_ = 1.438 Mg m^−^^3^
*M**_r_* = 647.18	Mo *K*α radiation, λ = 0.71073 Å
Orthorhombic, *F**d**d*2	Cell parameters from 5368 reflections
*a* = 21.673 (4) Å	θ = 2.4–29.7°
*b* = 28.296 (6) Å	µ = 0.76 mm^−^^1^
*c* = 9.7466 (19) Å	*T* = 83 K
*V* = 5977 (2) Å^3^	Pyramidal, colorless
*Z* = 8	0.35 × 0.27 × 0.17 mm
*F*(000) = 2672	

### Data collection

**Table:**

Bruker P4 diffractometer	4237 independent reflections
Parallel,graphite monochromator	4172 reflections with *I* > 2σ(*I*)
Detector resolution: 8.3 pixels mm^-1^	*R*_int_ = 0.025
ω scans	θ_max_ = 30.1°, θ_min_ = 2.4°
Absorption correction: multi-scan (SADABS; Bruker, 2002)	*h* = −29→29
*T*_min_ = 0.777, *T*_max_ = 0.882	*k* = −39→39
18986 measured reflections	*l* = −13→13

### Refinement

**Table:**

Refinement on *F*^2^	Hydrogen site location: inferred from neighbouring sites
Least-squares matrix: full	H-atom parameters constrained
*R*[*F*^2^ > 2σ(*F*^2^)] = 0.024	*w* = 1/[σ^2^(*F*_o_^2^) + (0.022*P*)^2^ + 9.9744*P*] where *P* = (*F*_o_^2^ + 2*F*_c_^2^)/3
*wR*(*F*^2^) = 0.055	(Δ/σ)_max_ = 0.001
*S* = 1.12	Δρ_max_ = 0.52 e Å^−^^3^
4237 reflections	Δρ_min_ = −0.53 e Å^−^^3^
253 parameters	Absolute structure: Flack *x* determined using 1892 quotients [(*I*^+^)-(*I*^-^)]/[(*I*^+^)+(*I*^-^)] (Parsons *et al.*, 2013)
319 restraints	Absolute structure parameter: −0.001 (16)
Primary atom site location: structure-invariant direct methods	

### Special details

**Table:**

Experimental. The data collection nominally covered a full sphere of reciprocal space by a combination of 5 sets of ω scans each set at different φ and/or 2θ angles and each scan (5 s exposure) covering -0.300° degrees in ω. The crystal to detector distance was 5.035 cm.
Geometry. All esds (except the esd in the dihedral angle between two l.s. planes) are estimated using the full covariance matrix. The cell esds are taken into account individually in the estimation of esds in distances, angles and torsion angles; correlations between esds in cell parameters are only used when they are defined by crystal symmetry. An approximate (isotropic) treatment of cell esds is used for estimating esds involving l.s. planes.
Refinement. Each Cp was disordered and modelled over two positions with 50% occupancy with restraints (SIMU).

### Fractional atomic coordinates and isotropic or equivalent isotropic displacement parameters (Å^2^)

**Table:**

	*x*	*y*	*z*	*U*_iso_*/*U*_eq_	Occ. (<1)
C1	0.4149 (3)	0.1012 (2)	0.1240 (7)	0.0231 (10)	0.5
H1	0.429216	0.120919	0.051940	0.028*	0.5
C2	0.4512 (10)	0.0654 (5)	0.197 (2)	0.0246 (15)	0.5
H2	0.493233	0.057360	0.181473	0.030*	0.5
C3	0.4115 (4)	0.0457 (2)	0.2919 (7)	0.0264 (10)	0.5
H3	0.422729	0.021677	0.355079	0.032*	0.5
C4	0.3518 (6)	0.0661 (3)	0.2832 (11)	0.0264 (14)	0.5
H4	0.316544	0.057877	0.336223	0.032*	0.5
C5	0.3553 (3)	0.1011 (2)	0.1793 (7)	0.0266 (10)	0.5
H5	0.322527	0.121326	0.151809	0.032*	0.5
C6	0.3122 (7)	0.0244 (3)	−0.1688 (15)	0.0269 (16)	0.5
H6	0.334822	0.029135	−0.251210	0.032*	0.5
C7	0.2998 (5)	−0.0191 (3)	−0.1095 (13)	0.0272 (15)	0.5
H7	0.313560	−0.048819	−0.142771	0.033*	0.5
C8	0.2638 (4)	−0.0119 (3)	0.0073 (12)	0.0260 (15)	0.5
H8	0.247994	−0.036139	0.064640	0.031*	0.5
C9	0.2547 (5)	0.0358 (3)	0.0267 (13)	0.0300 (16)	0.5
H9	0.232528	0.050052	0.099854	0.036*	0.5
C10	0.2859 (5)	0.0607 (4)	−0.0871 (12)	0.0306 (15)	0.5
H10	0.287840	0.093848	−0.102312	0.037*	0.5
C1'	0.3957 (4)	0.0989 (2)	0.1826 (8)	0.0294 (10)	0.5
H1'	0.389903	0.126670	0.129223	0.035*	0.5
C2'	0.4486 (9)	0.0729 (4)	0.191 (2)	0.0235 (15)	0.5
H2'	0.486339	0.080007	0.145464	0.028*	0.5
C3'	0.4379 (3)	0.0332 (2)	0.2784 (6)	0.0253 (11)	0.5
H3'	0.466623	0.009067	0.300743	0.030*	0.5
C4'	0.3769 (3)	0.0368 (2)	0.3251 (7)	0.0281 (10)	0.5
H4'	0.356658	0.015415	0.385330	0.034*	0.5
C5'	0.3514 (6)	0.0774 (3)	0.2672 (13)	0.0280 (15)	0.5
H5'	0.310669	0.088612	0.282472	0.034*	0.5
C6'	0.3136 (7)	0.0370 (4)	−0.1603 (15)	0.0280 (17)	0.5
H6'	0.337190	0.041941	−0.241098	0.034*	0.5
C7'	0.2922 (5)	−0.0066 (4)	−0.1119 (15)	0.0302 (15)	0.5
H7'	0.299610	−0.036621	−0.152015	0.036*	0.5
C8'	0.2570 (5)	0.0027 (4)	0.0094 (12)	0.0303 (16)	0.5
H8'	0.236792	−0.019994	0.065327	0.036*	0.5
C9'	0.2582 (5)	0.0507 (3)	0.0295 (14)	0.0315 (16)	0.5
H9'	0.237352	0.067093	0.100873	0.038*	0.5
C10'	0.2943 (5)	0.0715 (4)	−0.0698 (13)	0.0331 (17)	0.5
H10'	0.304285	0.104167	−0.075173	0.040*	0.5
C11	0.36287 (17)	−0.04990 (13)	0.1677 (4)	0.0385 (8)	
H11A	0.351416	−0.073878	0.099754	0.058*	
H11B	0.331532	−0.048788	0.240107	0.058*	
H11C	0.402937	−0.057999	0.207962	0.058*	
C12	0.51765 (14)	0.05586 (10)	−0.2559 (3)	0.0239 (6)	
C13	0.54483 (15)	0.09254 (10)	−0.1573 (3)	0.0289 (6)	
H13A	0.581961	0.079605	−0.113902	0.043*	
H13B	0.514340	0.100272	−0.086530	0.043*	
H13C	0.555584	0.121209	−0.208403	0.043*	
C14	0.56515 (18)	0.04516 (12)	−0.3690 (3)	0.0364 (8)	
H14A	0.578096	0.074766	−0.412501	0.055*	
H14B	0.546486	0.024413	−0.437995	0.055*	
H14C	0.601131	0.029476	−0.328548	0.055*	
C15	0.46013 (17)	0.07800 (10)	−0.3227 (3)	0.0304 (7)	
H15A	0.430632	0.087027	−0.251124	0.046*	
H15B	0.440912	0.054981	−0.384471	0.046*	
H15C	0.472226	0.106096	−0.374981	0.046*	
O1	0.43966 (9)	0.01044 (7)	−0.0586 (2)	0.0188 (4)	
Si1	0.500000	0.000000	−0.15337 (9)	0.01599 (18)	
Zr1	0.36931 (2)	0.02294 (2)	0.06245 (3)	0.01769 (7)	

### Atomic displacement parameters (Å^2^)

**Table:**

	*U*^11^	*U*^22^	*U*^33^	*U*^12^	*U*^13^	*U*^23^
C1	0.027 (2)	0.019 (2)	0.024 (2)	−0.0031 (19)	0.004 (2)	−0.0088 (19)
C2	0.026 (2)	0.025 (3)	0.022 (2)	−0.001 (3)	−0.005 (2)	−0.006 (3)
C3	0.033 (2)	0.028 (2)	0.019 (2)	−0.003 (2)	−0.004 (2)	−0.0031 (18)
C4	0.031 (2)	0.027 (3)	0.021 (3)	−0.007 (3)	0.006 (2)	−0.006 (3)
C5	0.028 (2)	0.023 (2)	0.029 (2)	0.000 (2)	0.002 (2)	−0.0147 (19)
C6	0.023 (2)	0.032 (4)	0.025 (2)	0.004 (3)	−0.010 (2)	−0.001 (3)
C7	0.021 (3)	0.031 (4)	0.029 (2)	0.001 (2)	−0.011 (2)	−0.004 (3)
C8	0.016 (2)	0.027 (4)	0.034 (2)	−0.001 (2)	−0.004 (2)	−0.005 (3)
C9	0.016 (2)	0.036 (4)	0.037 (2)	0.003 (3)	−0.0038 (19)	−0.003 (3)
C10	0.022 (3)	0.035 (4)	0.035 (3)	0.003 (2)	−0.013 (2)	−0.002 (3)
C1'	0.036 (2)	0.022 (2)	0.030 (2)	−0.005 (2)	−0.001 (2)	−0.005 (2)
C2'	0.028 (3)	0.021 (3)	0.021 (2)	−0.009 (3)	0.001 (2)	−0.005 (3)
C3'	0.027 (2)	0.029 (2)	0.020 (2)	0.002 (2)	−0.005 (2)	−0.006 (2)
C4'	0.031 (2)	0.032 (2)	0.022 (2)	−0.007 (2)	0.005 (2)	−0.004 (2)
C5'	0.031 (2)	0.025 (3)	0.028 (3)	0.003 (3)	0.003 (2)	−0.008 (2)
C6'	0.021 (2)	0.036 (4)	0.027 (3)	0.000 (3)	−0.010 (2)	0.006 (3)
C7'	0.021 (2)	0.036 (4)	0.033 (2)	0.001 (3)	−0.011 (2)	−0.001 (3)
C8'	0.019 (2)	0.038 (4)	0.035 (2)	−0.002 (3)	−0.005 (2)	0.003 (3)
C9'	0.018 (2)	0.037 (4)	0.039 (3)	0.005 (3)	−0.004 (2)	0.000 (3)
C10'	0.023 (3)	0.037 (4)	0.040 (3)	0.008 (3)	−0.012 (2)	0.003 (3)
C11	0.0400 (19)	0.0380 (18)	0.0376 (19)	0.0013 (14)	0.0140 (15)	0.0159 (15)
C12	0.0353 (15)	0.0190 (12)	0.0175 (12)	0.0011 (11)	0.0031 (11)	0.0039 (10)
C13	0.0398 (16)	0.0215 (12)	0.0256 (15)	−0.0065 (11)	−0.0006 (12)	0.0056 (11)
C14	0.053 (2)	0.0285 (15)	0.0278 (16)	0.0030 (15)	0.0166 (15)	0.0103 (12)
C15	0.0468 (19)	0.0221 (13)	0.0224 (14)	0.0047 (13)	−0.0058 (13)	0.0029 (11)
O1	0.0199 (9)	0.0173 (8)	0.0193 (9)	0.0003 (7)	−0.0017 (7)	0.0000 (7)
Si1	0.0220 (4)	0.0152 (4)	0.0108 (4)	0.0005 (3)	0.000	0.000
Zr1	0.01532 (10)	0.01792 (10)	0.01984 (11)	0.00056 (9)	−0.00167 (9)	−0.00222 (9)

### Geometric parameters (Å, º)

**Table:**

C1—H1	0.9500	C4'—Zr1	2.595 (7)
C1—C2	1.465 (16)	C5'—H5'	0.9500
C1—C5	1.399 (9)	C5'—Zr1	2.550 (12)
C1—Zr1	2.497 (6)	C6'—H6'	0.9500
C2—H2	0.9500	C6'—C7'	1.398 (13)
C2—C3	1.38 (2)	C6'—C10'	1.381 (14)
C2—Zr1	2.51 (2)	C6'—Zr1	2.516 (14)
C3—H3	0.9500	C7'—H7'	0.9500
C3—C4	1.419 (15)	C7'—C8'	1.431 (15)
C3—Zr1	2.500 (6)	C7'—Zr1	2.525 (13)
C4—H4	0.9500	C8'—H8'	0.9500
C4—C5	1.420 (11)	C8'—C9'	1.373 (11)
C4—Zr1	2.503 (11)	C8'—Zr1	2.552 (11)
C5—H5	0.9500	C9'—H9'	0.9500
C5—Zr1	2.507 (6)	C9'—C10'	1.376 (15)
C6—H6	0.9500	C9'—Zr1	2.553 (11)
C6—C7	1.385 (13)	C10'—H10'	0.9500
C6—C10	1.421 (13)	C10'—Zr1	2.489 (12)
C6—Zr1	2.572 (14)	C11—H11A	0.9800
C7—H7	0.9500	C11—H11B	0.9800
C7—C8	1.395 (14)	C11—H11C	0.9800
C7—Zr1	2.547 (12)	C11—Zr1	2.307 (3)
C8—H8	0.9500	C12—C13	1.532 (4)
C8—C9	1.376 (10)	C12—C14	1.538 (4)
C8—Zr1	2.547 (10)	C12—C15	1.540 (4)
C9—H9	0.9500	C12—Si1	1.909 (3)
C9—C10	1.478 (15)	C13—H13A	0.9800
C9—Zr1	2.534 (11)	C13—H13B	0.9800
C10—H10	0.9500	C13—H13C	0.9800
C10—Zr1	2.557 (12)	C14—H14A	0.9800
C1'—H1'	0.9500	C14—H14B	0.9800
C1'—C2'	1.364 (19)	C14—H14C	0.9800
C1'—C5'	1.406 (15)	C15—H15A	0.9800
C1'—Zr1	2.514 (6)	C15—H15B	0.9800
C2'—H2'	0.9500	C15—H15C	0.9800
C2'—C3'	1.431 (17)	O1—Si1	1.628 (2)
C2'—Zr1	2.553 (19)	O1—Zr1	1.9599 (19)
C3'—H3'	0.9500	Zr1—Cp1	2.196
C3'—C4'	1.401 (9)	Zr1—Cp1'	2.258
C3'—Zr1	2.593 (6)	Zr1—Cp2	2.202
C4'—H4'	0.9500	Zr1—Cp2'	2.233
C4'—C5'	1.394 (11)		
			
C2—C1—H1	126.0	C12—C14—H14B	109.5
C2—C1—Zr1	73.5 (8)	C12—C14—H14C	109.5
C5—C1—H1	126.0	H14A—C14—H14B	109.5
C5—C1—C2	108.0 (10)	H14A—C14—H14C	109.5
C5—C1—Zr1	74.2 (3)	H14B—C14—H14C	109.5
Zr1—C1—H1	118.3	C12—C15—H15A	109.5
C1—C2—H2	127.3	C12—C15—H15B	109.5
C1—C2—Zr1	72.5 (8)	C12—C15—H15C	109.5
C3—C2—C1	105.5 (14)	H15A—C15—H15B	109.5
C3—C2—H2	127.3	H15A—C15—H15C	109.5
C3—C2—Zr1	73.6 (9)	H15B—C15—H15C	109.5
Zr1—C2—H2	118.9	Si1—O1—Zr1	177.55 (13)
C2—C3—H3	124.3	C12—Si1—C12^i^	116.86 (18)
C2—C3—C4	111.4 (9)	O1^i^—Si1—C12	106.64 (11)
C2—C3—Zr1	74.4 (9)	O1—Si1—C12^i^	106.64 (11)
C4—C3—H3	124.3	O1—Si1—C12	107.93 (11)
C4—C3—Zr1	73.6 (5)	O1^i^—Si1—C12^i^	107.92 (11)
Zr1—C3—H3	119.3	O1—Si1—O1^i^	110.86 (15)
C3—C4—H4	127.0	C1—Zr1—C2	34.0 (3)
C3—C4—C5	106.0 (9)	C1—Zr1—C3	54.0 (2)
C3—C4—Zr1	73.4 (5)	C1—Zr1—C4	54.6 (3)
C5—C4—H4	127.0	C1—Zr1—C5	32.5 (2)
C5—C4—Zr1	73.7 (5)	C1—Zr1—C6	112.8 (3)
Zr1—C4—H4	118.1	C1—Zr1—C7	143.7 (3)
C1—C5—C4	109.0 (8)	C1—Zr1—C8	138.5 (2)
C1—C5—H5	125.5	C1—Zr1—C9	107.1 (3)
C1—C5—Zr1	73.4 (3)	C1—Zr1—C10	92.6 (3)
C4—C5—H5	125.5	C2—Zr1—C6	142.1 (5)
C4—C5—Zr1	73.4 (5)	C2—Zr1—C7	169.7 (6)
Zr1—C5—H5	119.5	C2—Zr1—C8	158.4 (6)
C7—C6—H6	125.4	C2—Zr1—C9	134.1 (5)
C7—C6—C10	109.3 (10)	C2—Zr1—C10	126.7 (4)
C7—C6—Zr1	73.3 (7)	C3—Zr1—C2	32.0 (5)
C10—C6—H6	125.4	C3—Zr1—C4	33.0 (4)
C10—C6—Zr1	73.3 (6)	C3—Zr1—C5	53.9 (2)
Zr1—C6—H6	119.7	C3—Zr1—C6	162.9 (3)
C6—C7—H7	125.7	C3—Zr1—C7	157.6 (3)
C6—C7—C8	108.6 (8)	C3—Zr1—C8	128.1 (3)
C6—C7—Zr1	75.3 (7)	C3—Zr1—C9	116.4 (3)
C8—C7—H7	125.7	C3—Zr1—C10	131.3 (3)
C8—C7—Zr1	74.1 (5)	C4—Zr1—C2	55.0 (6)
Zr1—C7—H7	116.9	C4—Zr1—C5	32.9 (2)
C7—C8—H8	125.2	C4—Zr1—C6	132.3 (4)
C7—C8—Zr1	74.1 (5)	C4—Zr1—C7	134.7 (4)
C9—C8—C7	109.6 (8)	C4—Zr1—C8	103.5 (4)
C9—C8—H8	125.2	C4—Zr1—C9	84.2 (4)
C9—C8—Zr1	73.8 (6)	C4—Zr1—C10	100.3 (4)
Zr1—C8—H8	118.7	C5—Zr1—C2	55.0 (4)
C8—C9—H9	126.3	C5—Zr1—C6	109.0 (3)
C8—C9—C10	107.4 (8)	C5—Zr1—C7	129.7 (3)
C8—C9—Zr1	74.8 (6)	C5—Zr1—C8	109.2 (2)
C10—C9—H9	126.3	C5—Zr1—C9	79.5 (3)
C10—C9—Zr1	74.0 (5)	C5—Zr1—C10	78.7 (3)
Zr1—C9—H9	117.0	C7—Zr1—C6	31.4 (3)
C6—C10—C9	105.0 (8)	C7—Zr1—C10	53.3 (3)
C6—C10—H10	127.5	C8—Zr1—C6	52.4 (4)
C6—C10—Zr1	74.5 (7)	C8—Zr1—C7	31.8 (3)
C9—C10—H10	127.5	C8—Zr1—C10	53.6 (3)
C9—C10—Zr1	72.3 (6)	C9—Zr1—C6	53.5 (4)
Zr1—C10—H10	118.0	C9—Zr1—C7	52.9 (3)
C2'—C1'—H1'	126.1	C9—Zr1—C8	31.4 (2)
C2'—C1'—C5'	107.8 (10)	C9—Zr1—C10	33.7 (3)
C2'—C1'—Zr1	75.9 (8)	C10—Zr1—C6	32.2 (3)
C5'—C1'—H1'	126.1	C1'—Zr1—C2'	31.2 (4)
C5'—C1'—Zr1	75.3 (5)	C1'—Zr1—C5'	32.2 (3)
Zr1—C1'—H1'	114.9	C1'—Zr1—C6'	112.1 (3)
C1'—C2'—H2'	125.6	C1'—Zr1—C7'	138.3 (3)
C1'—C2'—C3'	108.7 (14)	C1'—Zr1—C8'	120.2 (3)
C1'—C2'—Zr1	72.8 (9)	C1'—Zr1—C9'	90.6 (3)
C3'—C2'—H2'	125.6	C2'—Zr1—C9'	121.7 (4)
C3'—C2'—Zr1	75.4 (8)	C5'—Zr1—C2'	52.0 (5)
Zr1—C2'—H2'	118.0	C5'—Zr1—C8'	98.6 (4)
C2'—C3'—H3'	126.5	C5'—Zr1—C9'	76.6 (4)
C2'—C3'—Zr1	72.3 (9)	C6'—Zr1—C2'	131.2 (4)
C4'—C3'—C2'	106.9 (10)	C6'—Zr1—C5'	120.4 (4)
C4'—C3'—H3'	126.5	C6'—Zr1—C7'	32.2 (3)
C4'—C3'—Zr1	74.4 (4)	C6'—Zr1—C8'	53.3 (4)
Zr1—C3'—H3'	118.8	C6'—Zr1—C9'	52.4 (4)
C3'—C4'—H4'	126.2	C7'—Zr1—C2'	163.4 (5)
C3'—C4'—Zr1	74.2 (4)	C7'—Zr1—C5'	128.7 (4)
C5'—C4'—C3'	107.6 (8)	C7'—Zr1—C8'	32.7 (3)
C5'—C4'—H4'	126.2	C7'—Zr1—C9'	52.6 (3)
C5'—C4'—Zr1	72.5 (6)	C8'—Zr1—C2'	149.9 (5)
Zr1—C4'—H4'	118.9	C8'—Zr1—C9'	31.2 (3)
C1'—C5'—H5'	125.5	C10'—Zr1—C1'	85.3 (3)
C1'—C5'—Zr1	72.5 (6)	C10'—Zr1—C2'	112.8 (4)
C4'—C5'—C1'	108.9 (10)	C10'—Zr1—C5'	88.4 (4)
C4'—C5'—H5'	125.5	C10'—Zr1—C6'	32.0 (3)
C4'—C5'—Zr1	76.1 (6)	C10'—Zr1—C7'	53.3 (3)
Zr1—C5'—H5'	117.8	C10'—Zr1—C8'	52.9 (3)
C7'—C6'—H6'	126.0	C10'—Zr1—C9'	31.6 (3)
C7'—C6'—Zr1	74.3 (7)	C11—Zr1—C1	135.18 (19)
C10'—C6'—H6'	126.0	C11—Zr1—C2	103.8 (4)
C10'—C6'—C7'	107.9 (10)	C11—Zr1—C3	81.65 (19)
C10'—C6'—Zr1	72.9 (7)	C11—Zr1—C4	92.5 (2)
Zr1—C6'—H6'	118.7	C11—Zr1—C5	125.4 (2)
C6'—C7'—H7'	126.5	C11—Zr1—C6	112.0 (3)
C6'—C7'—C8'	107.1 (9)	C11—Zr1—C7	80.8 (3)
C6'—C7'—Zr1	73.5 (7)	C11—Zr1—C8	72.2 (2)
C8'—C7'—H7'	126.5	C11—Zr1—C9	97.5 (2)
C8'—C7'—Zr1	74.7 (6)	C11—Zr1—C10	125.8 (3)
Zr1—C7'—H7'	117.5	C11—Zr1—C1'	124.8 (2)
C7'—C8'—H8'	126.6	C11—Zr1—C2'	108.6 (4)
C7'—C8'—Zr1	72.6 (6)	C11—Zr1—C5'	100.5 (2)
C9'—C8'—C7'	106.8 (9)	C11—Zr1—C6'	119.7 (3)
C9'—C8'—H8'	126.6	C11—Zr1—C7'	87.9 (3)
C9'—C8'—Zr1	74.4 (6)	C11—Zr1—C8'	80.3 (3)
Zr1—C8'—H8'	118.4	C11—Zr1—C9'	105.9 (2)
C8'—C9'—H9'	125.3	C11—Zr1—C10'	133.2 (3)
C8'—C9'—C10'	109.5 (9)	O1—Zr1—C1	89.83 (16)
C8'—C9'—Zr1	74.4 (6)	O1—Zr1—C2	81.4 (5)
C10'—C9'—H9'	125.3	O1—Zr1—C3	107.49 (19)
C10'—C9'—Zr1	71.6 (6)	O1—Zr1—C4	136.4 (3)
Zr1—C9'—H9'	120.5	O1—Zr1—C5	121.80 (16)
C6'—C10'—H10'	125.7	O1—Zr1—C6	81.3 (3)
C6'—C10'—Zr1	75.1 (7)	O1—Zr1—C7	88.8 (3)
C9'—C10'—C6'	108.6 (9)	O1—Zr1—C8	120.1 (2)
C9'—C10'—H10'	125.7	O1—Zr1—C9	134.9 (3)
C9'—C10'—Zr1	76.8 (6)	O1—Zr1—C10	106.4 (3)
Zr1—C10'—H10'	114.6	O1—Zr1—C1'	104.9 (2)
H11A—C11—H11B	109.5	O1—Zr1—C2'	82.6 (5)
H11A—C11—H11C	109.5	O1—Zr1—C5'	134.3 (3)
H11B—C11—H11C	109.5	O1—Zr1—C6'	83.3 (3)
Zr1—C11—H11A	109.5	O1—Zr1—C7'	92.9 (3)
Zr1—C11—H11B	109.5	O1—Zr1—C8'	125.4 (3)
Zr1—C11—H11C	109.5	O1—Zr1—C9'	135.5 (3)
C13—C12—C14	109.0 (3)	O1—Zr1—C10'	107.2 (3)
C13—C12—C15	107.5 (2)	O1—Zr1—C11	98.83 (11)
C13—C12—Si1	108.04 (19)	O1—Zr1—Cp1	108.60
C14—C12—C15	108.6 (2)	O1—Zr1—Cp1'	109.30
C14—C12—Si1	110.3 (2)	O1—Zr1—Cp2	107.31
C15—C12—Si1	113.3 (2)	O1—Zr1—Cp2'	110.27
C12—C13—H13A	109.5	C11—Zr1—Cp1	109.36
C12—C13—H13B	109.5	C11—Zr1—Cp1'	96.81
C12—C13—H13C	109.5	C11—Zr1—Cp2	98.16
H13A—C13—H13B	109.5	C11—Zr1—Cp2'	106.27
H13A—C13—H13C	109.5	Cp1—Zr1—Cp2	130.26
H13B—C13—H13C	109.5	Cp1'—Zr1—Cp2'	129.80
C12—C14—H14A	109.5		
			
C1—C2—C3—C4	1.0 (15)	C6'—C7'—C8'—C9'	0.2 (11)
C1—C2—C3—Zr1	65.9 (10)	C6'—C7'—C8'—Zr1	−66.9 (8)
C2—C1—C5—C4	−1.0 (10)	C7'—C6'—C10'—C9'	−3.6 (12)
C2—C1—C5—Zr1	−66.3 (8)	C7'—C6'—C10'—Zr1	66.6 (8)
C2—C3—C4—C5	−1.6 (13)	C7'—C8'—C9'—C10'	−2.4 (11)
C2—C3—C4—Zr1	65.4 (10)	C7'—C8'—C9'—Zr1	−65.9 (7)
C3—C4—C5—C1	1.6 (9)	C8'—C9'—C10'—C6'	3.8 (12)
C3—C4—C5—Zr1	66.8 (6)	C8'—C9'—C10'—Zr1	−65.2 (7)
C5—C1—C2—C3	0.0 (14)	C10'—C6'—C7'—C8'	2.1 (12)
C5—C1—C2—Zr1	66.7 (6)	C10'—C6'—C7'—Zr1	−65.7 (8)
C6—C7—C8—C9	−2.2 (11)	Zr1—C1—C2—C3	−66.7 (10)
C6—C7—C8—Zr1	−68.0 (8)	Zr1—C1—C5—C4	65.2 (6)
C7—C6—C10—C9	−1.3 (11)	Zr1—C2—C3—C4	−64.9 (7)
C7—C6—C10—Zr1	65.1 (8)	Zr1—C3—C4—C5	−67.0 (6)
C7—C8—C9—C10	1.3 (10)	Zr1—C4—C5—C1	−65.2 (5)
C7—C8—C9—Zr1	−66.0 (7)	Zr1—C6—C7—C8	67.2 (7)
C8—C9—C10—C6	0.0 (10)	Zr1—C6—C10—C9	−66.3 (7)
C8—C9—C10—Zr1	−67.9 (7)	Zr1—C7—C8—C9	65.8 (7)
C10—C6—C7—C8	2.1 (12)	Zr1—C8—C9—C10	67.4 (6)
C10—C6—C7—Zr1	−65.1 (8)	Zr1—C9—C10—C6	67.9 (7)
C1'—C2'—C3'—C4'	−1.2 (15)	Zr1—C1'—C2'—C3'	−67.5 (11)
C1'—C2'—C3'—Zr1	65.8 (11)	Zr1—C1'—C5'—C4'	68.0 (7)
C2'—C1'—C5'—C4'	−1.7 (14)	Zr1—C2'—C3'—C4'	−66.9 (6)
C2'—C1'—C5'—Zr1	−69.6 (10)	Zr1—C3'—C4'—C5'	−65.4 (7)
C2'—C3'—C4'—C5'	0.1 (12)	Zr1—C4'—C5'—C1'	−65.6 (7)
C2'—C3'—C4'—Zr1	65.5 (9)	Zr1—C6'—C7'—C8'	67.7 (7)
C3'—C4'—C5'—C1'	0.9 (11)	Zr1—C6'—C10'—C9'	−70.1 (8)
C3'—C4'—C5'—Zr1	66.5 (5)	Zr1—C7'—C8'—C9'	67.1 (7)
C5'—C1'—C2'—C3'	1.7 (16)	Zr1—C8'—C9'—C10'	63.5 (7)
C5'—C1'—C2'—Zr1	69.2 (8)	Zr1—C9'—C10'—C6'	69.0 (8)

Symmetry code: (i) −*x*+1, −*y*, *z*.
