# Supplementary material for: (μ-Di-tert-butyl­silanediolato)bis­[bis­(η5-cyclo­penta­dien­yl)methyl­zirconium]
Source: Acta Crystallogr E Crystallogr Commun. 2019 Nov 8;75(Pt 12):1848–52. doi: 10.1107/S2056989019014762 (PMC6895932; doi:10.1107/S2056989019014762)
Supplement: Supplementary file 3 [file e-75-01848-sup3.docx]

**Refcodes and literature citations for crystal structures with Cp_2_Zr-CH_3_ units for Zr-C distance comparisons**

AQESIZ Mukherjee, A., Sharanappa, N., Sen, T. K., Sarish, S. P., Ghorai, P. K., Ott, H., Stalke, D., Mandal, S. K. & Roesky, H. W. (2011) *Angew. Chem., Int. Ed.* **50**, 3968.

AXIBOA Boulho, C., Zijlstra, H. S., Hofmann, A., Budzelaar, P. H. M & Harder, S. (2016) *Chem.-Eur. J.* **22**, 17450.

BESGOW Bolig, A. D. & Chen, E. X. Y. (2004) *J. Am. Chem. Soc.* **126**, 4897.

BODMIR Helmstedt, U., Lebedkin, S., Hocher, T., Blaurock, S. & Hey-Hawkins, E. (2008) *Inorg. Chem.* **47**, 5815.

BUHVAD Xu, X., Kehr, G., Daniliuc, C. G. & Erker, G. (2015) *Organometallics*, **34**, 2655.

BUYSOD10 Longato, B., Martin, B. D., Norton, J. R. & Anderson, O. P. (1985) *Inorg. Chem.* **24**, 1389.

CADRUU Hunter, W. E., Hrncir, D. C., Bynum, R. V., Penttila, R. A., Atwood, J. L. (1983) *Organometallics*, **2**, 750.

COHTEY Waymouth, R. W., Santarsiero, B. D. & Grubbs, R. H. (1984) *J. Am. Chem. Soc.* **106**, 4050.

COPRII Ho, S. C. H., Straus, D. A., Armantrout, J. A., Schaefer, W. P. & Grubbs, R. H. (1984) *J. Am. Chem. Soc.* **106**, 2210.

DAGKAX Gambarotta, S., Strologo, S., Floriani, C., Chiesi-Villa, A. & Guastini, C. (1985) *Inorg. Chem.* **24**, 654.

DAGKIF Gambarotta, S., Strologo, S., Floriani, C., Chiesi-Villa, A. & Guastini, C. (1985) *Inorg. Chem.* **24**, 654.

DITHAP Martin, B. D., Matchett, S. A., Norton, J. R. & Anderson, O. P. (1985) *J. Am. Chem. Soc.* **107**, 7952.

EHEFUT Neu, R. C., Otten, E., Lough, A. & Stephan, D. W. (2011) *Chemical Science* **2**, 170.

EKEVEX Normand, A. T., Daniliuc, C. G., Wibbeling, B., Kehr, G., Le Gendre, P. & Erker, G. (2016) *Chem.-Eur. J.* **22**, 4285.

EKEVIB Normand, A. T., Daniliuc, C. G., Wibbeling, B., Kehr, G., Le Gendre, P. & Erker, G. (2016) *Chem.-Eur. J.* **22**, 4285.

EKEVOH Normand, A. T., Daniliuc, C. G., Wibbeling, B., Kehr, G., Le Gendre, P. & Erker, G. (2016) *Chem.-Eur. J.* **22**, 4285.

EKEVUN Normand, A. T., Daniliuc, C. G., Wibbeling, B., Kehr, G., Le Gendre, P. & Erker, G. (2016) *Chem.-Eur. J.* **22**, 4285.

EKEWAU Normand, A. T., Daniliuc, C. G., Wibbeling, B., Kehr, G., Le Gendre, P. & Erker, G. (2016) *Chem.-Eur. J.* **22**, 4285.

EKEWEY Normand, A. T., Daniliuc, C. G., Wibbeling, B., Kehr, G., Le Gendre, P. & Erker, G. (2016) *Chem.-Eur. J.* **22**, 4285.

ESISAA Zuccaccia, C., Stahl, N. G., Macchioni, A., Chen,, M. C., Roberts, J. A. & Marks, T. J. (2004) *J. Am. Chem. Soc.* **126**, 1448.

GIPYUZ Matchett, S. A., Norton, J. P. & Anderson, O. P. (1988) *Organometallics* **7**, 2228.

HEMCOR Askham, F. R., Carroll, K. M., Briggs, P. M., Rheingold, A. L. & Haggerty, B. S. (1994) *Organometallics* **13**, 2139.

HIKHUF Gurubasavaraj, P. M., Roesky, H. W., Sharma, P. M. V., Oswald, R. B., Dolle, V., Herbst-Irmer, R. & Pal, A. (2007) *Organometallics* **26**, 3346.

HIKJAN Gurubasavaraj, P. M., Roesky, H. W., Sharma, P. M. V., Oswald, R. B., Dolle, V., Herbst-Irmer, R. & Pal, A. (2007) *Organometallics* **26**, 3346.

HUVLAL Fujdala, K. L., Oliver, A. G., Hollander, F. J., & Tilley, T. D. (2003) *Inorg. Chem.* **42**, 1140.

IGUDOD Huerlander, D., Kleigrewe, N., Kehr, G., Erker, G. & Frohlich, R. (2002) *Eur. J. Inorg. Chem.* p. 2633.

JITVAK Mandal, S. K., Gurubasavaraj, P. M., Roesky, H. W., Schwab, G., Stalke, D., Oswald, R. B. & Dolle, V. (2007) *Inorg. Chem.* **46** 10158.

JITVEO Mandal, S. K., Gurubasavaraj, P. M., Roesky, H. W., Schwab, G., Stalke, D., Oswald, R. B. & Dolle, V. (2007) *Inorg. Chem.* **46** 10158.

JUGCIZ Boulho, C., Zijlstra, H. S. & Harder, S. (2015) *Eur. J. Inorg. Chem.* p. 2132.

KEXYER Erker, G., Albrecht, M., Werner, S. & Kruger, C. (1990) *Z. Naturforsch., B: Chem. Sci.* **45**, 1205.

KODQAV Koch, T., Blaurock, S., Hey-Hawkins, E., Galan-Fereres, M., Plat, D. & Eisen, M. S. (2000) *J. Organomet. Chem.* **595**, 126.

KUPQAP Mariott, W. R. & Chen, E. Y. X. (2005) *Macromolecules* **38**, 6822.

LEDBEB Askham, F. R., Carroll, K. M., Alexander, S. J., Rheingold, A. L. & Haggerty, B. S. (1993) *Organometallics* **12**, 4810.

LEPXAH Mukherjee, A., Sen, T. K. Mandal, S. K., Maity, B. & Koley, D. (2013) *RSC Advances* **3**, 1255.

MOJHEZ Black, K., Aspinall, H. C., Jones, A. C., Przybylak, K., Bacsa, J., Chalker, P. R., Taylor, S., Zhao, C. Z., Elliot, S. D., Zydor, A. & Heys, P. N. (2008) *J. Mater. Chem.* **18**, 4561.

NAHYOL Bai, G., Singh, S., Roesky, H. W., Noltemeyer, M. & Schmidt, H.-G. (2005) *J. Am. Chem. Soc.* **127**, 3449.

NAPXUY Pineda, L. W., Jancik, V., Roesky, H. W. & Herbst-Irmer, R. (2005) *Inorg. Chem.* **44**, 3537.

NIMNOM Johnson, M. J. A., Odom, A. L. & Cummins, C. C. (1997) *Chem. Commun.* p. 1523.

ODOBIU Fromel, S., Kehr, G., Frohlich, R., Daniliuc, C. G. & Erker, G. (2013) *Dalton Trans.* **42**, 14531.

ODOBOA Fromel, S., Kehr, G., Frohlich, R., Daniliuc, C. G. & Erker, G. (2013) *Dalton Trans.* **42**, 14531.

ODOBUG Fromel, S., Kehr, G., Frohlich, R., Daniliuc, C. G. & Erker, G. (2013) *Dalton Trans.* **42**, 14531.

OKUFUX Bestgen, S., Schoo, C., Zovko, C., Koppe, R., Kelly, R. P., Lebedkin, S., Kappes, M. M. & Roesky, P. W. (2016) *Chem.-Eur. J.* **22**, 7115.

OZUCAO Kelsen, V., Vallee, C., Jeanneau, E., Bibal, C., Santini, C. C., Chauvin, Y. & Olivier-Bourbigou, H. (2011) *Organometallics* **30**, 4284.

PEDFUA Singh, S., Jancik, V., Roesky, H. W. & Herbst-Irmer, R. (2006)  *Inorg. Chem.* **45**, 949.

QIZCEI Yang, Y., Gurubasavaraj, P. M., Ye, H., Zhang, Z., Roesky, H. W. & Jones, P. G. (2008) *J. Organomet. Chem.* **693**, 1455.

REDTUQ Cummings, S. A., Radford, R., Erker, G. Kehr, G. & Frohlich, R. (2006) *Organometallics*, **25**, 839.

TIWKUG Yang, Y., Schulz, T., John, M., Yang, Z., Jimenez-Perez, V. M., Roesky, H. W., Gurubasavaraj, P. M., Stalke, D. & Ye, H. (2008) *Organometallics* **27**, 769.

TOWMUN Breen, T. L. & Stephan, D. W. (1996) *Organometallics* **15**, 4509.

VIBSOO Waymouth, R. W., Potter, K. S., Schaefer, W. P. & Grubbs, R. H. (1990) *Organometallics* **9**, 2843.

WAJLOJ Ruck, R. T. & Bergman, R. G. (2004) *Angew. Chem., Int. Ed.* **43**, 5375.

WATSOB Chapman, A. M., Haddow, M. F. & Wass, D. F. (2012) *Eur. J. Inorg. Chem.* p. 1546.

WATSUH Chapman, A. M., Haddow, M. F. & Wass, D. F. (2012) *Eur. J. Inorg. Chem.* p. 1546.

WATTAO Chapman, A. M., Haddow, M. F. & Wass, D. F. (2012) *Eur. J. Inorg. Chem.* p. 1546.

WAYMER Liu, Y.-L., Kehr, G., Daniliuc, C. G. & Erker, G. (2017) *Organometallics* **36**, 3407.

WETJEL Helmstedt, U., Lonnecke, P., Reinhold, J. & Hey-Hawkins, E. (2006) *Eur. J. Inorg. Chem.* p. 4922.

WEWRUO Jian, Z., Daniliuc, C. G., Kehr, G. & Erker, G. (2018) *Chem. Commun.* **54**, 5724.

WEXWED Nekoueishahraki, B., Jana, A., Roesky, H. W., Mishra, L., Stern, D. & Stalke, D. (2009) *Organometallics* **28**, 5733.

WUPVUA Gurubasavaraj, P. M. (2015) *CSD Communication* (Private Communication).

XESDEE Stuhldreier, T., Keul, H., Hocker, H. & Englert U. (2000) *Organometallics* **19**, 5231.

YIMKAG Ciruelo, G., Cuenca, T., Gomez-Sal, P., Martin, A. & Royo, P. *J. Chem. Soc., Dalton Trans.* p. 231.

**Refcodes and literature citations for crystal structures with Cp_2_Zr-O-Si units for Zr-O and O-Si distance comparisons**

EXUBII Garrison, J. C., Kim, H., Collins, S. & Youngs, W. J. (2004) *Acta Crystallogr., Sect. C: Cryst. Struct. Commun.* **60**, m357.

HECZEU Samuel, E., Harrod, J. F., McGlinchey, M. J., Cabestaing, C. & Robert, F. (1994) *Inorg. Chem.* **33**, 1292.

JANYEF Richers, C. P., Bertke, J. A. & Rauchfuss, T. B. (2017) *Dalton Trans.* **46**, 8756.

LEJSEZ Burlakov, V. V., Arndt, P., Baumann, W., Spannenberg, A. & Rosenthal, U. (2006) *Organometallics* **25**, 1317.

QAMLEW Wada, K., Itayama, N., Watanabe, N., Bundo, M., Kondo, T. & Mitsudo, T. *Organometallics* **23**, 5824.

REWKIN Abrahams, I., Simon, C., Motevalli,, M., Shah, S. A. A. & Sullivan, A. C. (1996) *J. Organomet. Chem.* **521**, 301.

ROCWIP Enders, M., Fink, J., Maillant, V. & Pritzkow, H. (2001) *Z. Anorg. Allg. Chem.* **627**, 2281.

TUDQEP Zhang, W., Zhang, S., Sun, X., Nishiura, M., Hou, Z. & Xi, Z. (2009) *Angew. Chem., Int. Ed.* **48**, 7227.

UGINIH Hofmann, M., Malisch, W., Schumacher, D., Lager, M. & Nieger, M. (2002) *Organometallics* **21**, 3485.

UGINON Hofmann, M., Malisch, W., Schumacher, D., Lager, M. & Nieger, M. (2002) *Organometallics* **21**, 3485.

UMOWUO Lacroix, F., Plecnik, C. E., Liu, S., Liu, F., Meyers, E. A. & Shore, S. G. (2003) *J. Organomet. Chem.* **687**, 69.

VAQMEH Varga, V., Horacek, M., Bastl, Z., Merna, J. Cisarova, I., Sykora, J. & Pinkas, J. (2012) *Catal. Today* **179**, 130.

WUSWAI Thieme, K., Bourke, S. C., Zheng, J., MacLachlan, M. J., Zamanian, F., Lough, A. J. & Manners, I. (2002) *Can. J. Chem.* **80**, 1469.

WUSWEM Thieme, K., Bourke, S. C., Zheng, J., MacLachlan, M. J., Zamanian, F., Lough, A. J. & Manners, I. (2002) *Can. J. Chem.* **80**, 1469.

XIXDIR Skowronska-Ptasinska, M. D., Duchateau, R., van Santen, R. A. & Yap, G. P. A. (2001) *Organometallics* **20**, 3519.

**Refcodes and literature citations for crystal structures with M-O-Si(*t*-Bu)_2_-O-M units for O-Si-O angle comparisons**

HETRED Gosink, H.-J., Roesky, H. W., Schmidt, H.-G., Noltemeyer, M., Irmer, E. & Herbst-Irmer, R. (1994) *Organometallics* **13**, 3420.

HETRON Gosink, H.-J., Roesky, H. W., Schmidt, H.-G., Noltemeyer, M., Irmer, E. & Herbst-Irmer, R. (1994) *Organometallics* **13**, 3420.

JIYBEY Roesky, H. W., Hesse, D., Bohra, R. & Noltemeyer, M. (1991) *Chem. Ber.* **124**, 1913.

KIPGUL Roesky, H. W., Mazzah, A., Hesse, D. & Noltemeyer, M. (1991) *Chem. Ber.* **124**, 519.

NADDAX Liu, F.-Q., Uson, I. & Roesky, H. W. (1996) *Z. Anorg. Allg. Chem.* **622**, 819.

PAHZED Liu, F.-Q., Schmidt, H.-G., Noltemeyer, M., Freire-Erdbrugger, C., Sheldrick, G. M. & Roesky, H. W. (1992) *Z. Naturforsch., B:*

*Chem. Sci.* **47**, 1085.

TAJYOS Haoudi-Mazzah, A., Mazzah, A., Schmidt, H.-G., Noltemeyer, M. & Roesky, H. W. (1991) *Z. Naturforsch., B: Chem. Sci.* **46**, 587.

TAJYUY Haoudi-Mazzah, A., Mazzah, A., Schmidt, H.-G., Noltemeyer, M. & Roesky, H. W. (1991) *Z. Naturforsch., B: Chem. Sci.* **46**, 587.

TAJZAF Haoudi-Mazzah, A., Mazzah, A., Schmidt, H.-G., Noltemeyer, M. & Roesky, H. W. (1991) *Z. Naturforsch., B: Chem. Sci.* **46**, 587.

VUMNUM Liu, F.-Q., Roesky, H. W., Schmidt, H.-G. & Noltemeyer, M. (1992) *Organometallics* **11**, 2965.

WAGVIJ Gosink, H.-J., Roesky, H. W., Noltemeyer, M., Schmidt, H.-G., Freire-Erdbrugger, C. & Sheldrick, G. M. (1993) *Chem. Ber.* **126**, 279.

WAGVOP Gosink, H.-J., Roesky, H. W., Noltemeyer, M., Schmidt, H.-G., Freire-Erdbrugger, C. & Sheldrick, G. M. (1993) *Chem. Ber.* **126**, 279.

ZEKKAB Liu, F.-Q., Uson, I. & Roesky, H. W. (1995) *J. Chem. Soc., Dalton Trans.* p. 2453.

ZEKKEF Liu, F.-Q., Uson, I. & Roesky, H. W. (1995) *J. Chem. Soc., Dalton Trans.* p. 2453.
